# Measurement of foliar H_2_O_2_ concentration can be an indicator of riparian vegetation management

**DOI:** 10.1038/s41598-022-17658-2

**Published:** 2022-08-13

**Authors:** Takashi Asaeda, Mizanur Rahman, Lekkala Vamsi-Krishna, Jonas Schoelynck, Md Harun Rashid

**Affiliations:** 1 Saitama University, Saitama, 338-8570 Japan; 2 Hydro Technology Institute, Shimo-meguro, Tokyo, Japan; 3 Research and Development Center, Nippon Koei, Tsukuba, Japan; 4 Diamonds Company, New Delhi, India; 5 University of Antwerp, ECOSPHERE Research Group, Wilrijk, Belgium; 6 Bangladesh Agricultural University, Mymensingh, Bangladesh

**Keywords:** Plant sciences, Plant ecology

## Abstract

Riparian vegetation is frequently exposed to abiotic stress, which generates reactive oxygen species (ROS) caused by strong differences in a river’s hydrological conditions. Among different ROS, hydrogen peroxide (H_2_O_2_) is relatively steady and can be measured appropriately. Thus, the quantification of plant H_2_O_2_ can be used as a stress indicator for riparian vegetation management. The current study examines the spatial distribution of plants by riparian vegetation communities across the elevation gradient of riparian zones through quantification of environmental stress using foliar H_2_O_2_ concentration. The trees *Salix* spp*.*, *Robinia pseudoacacia*, *Ailanthus altissima* with *Juglans mandshurica*, and the herbs *Phragmites australis*, *Phragmites japonica*, and *Miscanthus sacchariflorus* were selected for this study. Leaf tissues were collected to analyze H_2_O_2_ concentration, meanwhile riparian soil was sampled to measure total nitrogen (TN), total phosphorus (TP), and moisture content. The H_2_O_2_ concentration of tree species increased with higher soil moisture content, which was negatively correlated for *Salix* and herb spp., in which H_2_O_2_ concentration always decreased with high soil moisture. In this study, we found a unique significant interaction between soil moisture content and H_2_O_2_ concentration, both positively or negatively correlated relationships, when compared with other parameters, such as TN or TP concentrations or TN: TP in riparian soil. The species-specific distribution zones can be explained by the H_2_O_2_ concentration in the plant for gravelly and sandy channels on a theoretical range of soil moisture. Each species’ H_2_O_2_ concentration was estimated through derived equations and is directly related to an elevation above the channel. The comparison with the observed distribution of plant elevations in the field indicated that all species showed a spatial distribution that acts as species-specific elevations where H_2_O_2_ concentrations stayed below 40 μmol/gFW. Hence, the present study suggests that foliar H_2_O_2_ concentration can be a useful benchmark for the distribution potentiality of riparian vegetation.

## Introduction

Riparian vegetation is naturally adapted to abiotic conditions characterized by fluctuating water, sediment, and nutrients^1^. Riparian plants are diverse in species, structure, and regeneration strategies^2,3^. Therefore, riparian habitats are regarded as biodiversity corridors for restoration^4–7^, providing an ecotone between the terrestrial and aquatic ecosystems^8,9^. Despite the importance of riparian vegetation, riparian degradation often occurs due to various natural disturbances and human activities reducing species diversity^10,11^. This degradation affects the composition and the plant community structure^12^. The magnitude, frequency, and duration of flood events have a distinct impact on the creation of the riparian environment. This impact occurs through inundation frequency, level, duration^13–17^, and flow velocity during the inundated period, driving erosion in other fluvial landforms and deposition of the transported sediment^17–19^. The sediment moisture content of riparian zones is associated with the stratigraphy of alluvium, groundwater, hyporheic flows^20^, and the position in the flood or plain corridor^21^. However, it generally decreases with elevation, depending on the sediment particle size^22^. These conditions help a riparian species to grow at the elevation of its preferred riparian soil moisture zone^23^. Due to frequent flood disturbances the sediment accumulations cannot develop properly. As a result, its nutrient level may decrease^24,25^. The sediment nutrient level may also determine which species can distribute at specific zones^15,26,27^. The sediment nutrient level in riparian soil, especially TN or TP concentration, can filter and alter its biogeochemistry^28–31^. For instance, wetland plants take up 16–75% of TN^32,33^.

The species distribution depends on previously experienced abiotic stresses. However, when subjected to flood disturbance, river habitat conditions change frequently and often suddenly, which is related to the source of deposited sediment and fluvial dynamics. Therefore, it is difficult to evaluate the spatial distribution of each species. There may be an effect with the presence and abundance of dominent factors in preference to promote or inhibit under these complex and constantly changing stressful environments. Causal observation of plant traits, such as growth rate and biomass, commonly used in vegetation monitoring^34–36^, are not necessarily appropriate evaluation methods. The traits may have been developed under different abiotic conditions than the ones at the monitoring time. Thus, false correlations with the prevailing abiotic conditions may occur. Therefore, an immediate monitoring system is needed to gain information on the possible suitable conditions that can explain the spatial distribution of each species.

Living organisms and biological systems play a significant role against stress to prevent or repair damage. When plants are subjected to environmental stress through metabolic and physiological adjustments, ROS is generated in different organelles depending on the stressor types (e.g., anoxia, drought)^37,38^. Some ROS is scavenged relatively quickly by antioxidants, and the homogeneity of ROS in tissues is maintained by balancing the ROS and antioxidants. The balance flips over when oxidative stress surpasses the scavenging capacity of the antioxidants^39^. During exposure to different types of environmental stressors, H_2_O_2_ is generated^38,40^. The H_2_O_2_ in plant tissues is relatively stable and can easily be measured^41,42^. H_2_O_2_ measurement is suitable with minimum losses compared with other ROS, such as the superoxide radical (O_2_^·−^) and the hydroxyl radical (OH^·−^). H_2_O_2_ has been extensively used to quantify ROS damage or stress levels in many plant studies. Therefore, H_2_O_2_ can be used as an indicator of the physiological status of plants and to monitor the response of plants against the intensity of environmental stress^43–46^. The application of stress response biomarkers, such as H_2_O_2_ content in plant tissues using an empirical model, could be an efficient tool in the context of habitat suitability, too. It is necessary to determine the relationship between H_2_O_2_ concentration and species-specific abiotic conditions of its habitat to apply this H_2_O_2_ indicator in practice^47,48^.

The main objective of this study is to empirically determine the relationships between species-specific habitat conditions and the H_2_O_2_ concentration in riparian plant leaves to understand the feasible H_2_O_2_ level for the species to grow, in order to explore the conditions as an index of plant distribution.

## Results

### Edaphic condition of the riparian zone

**Figure 1:**
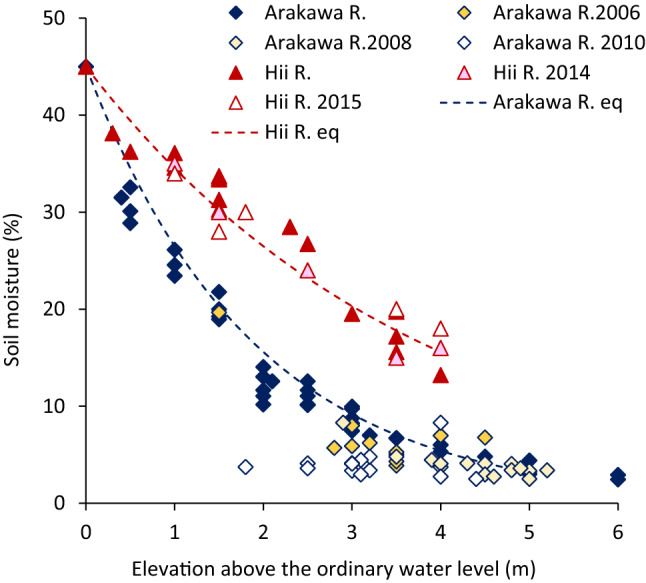
Relationship between elevation above ordinary water level and soil moisture. Data are from this study and previous studies from 2006 to 2015^22,30,56,66^.

The soil moisture content gradually decreased with increased elevation away from the channel at both the Arakawa River and Hii River sites (*r* = −0.90, *p* < 0.01 for the Arakawa River and *r* = −0.98, *p* < 0.01 for the Hii River) (Fig. 1). The distributions were empirically given as follows:

For the Arakawa River (gravelly) site (*r* = 0.99):

1 $${\text{Soil}}\,{\text{moisture}}\,\left( {\text{\%}}\right) = 45.0\,\exp. \left( { - 0.53\, {\text{elevation}}} \right)$$

For the Hii River (sandy) site (*r* = 0.99):

2 $${\text{Soil}}\,{\text{moisture}}\,\left( {\text{\%}} \right) = 45.0\,\exp. \left( { - 0.265\,{\text{elevation}}} \right).$$

where elevation is given in meters above the ordinary water level.

No significant difference was observed in the deviation from these curves between the previously observed results in other studies and the present study (Arakawa River, *r* = 0.91, *p* < 10^−7^ for 2006, *r* = 0.89, *p* < 10^−7^ for 2008, and *r* = 0.78, < 10^−7^ for 2010; Hii River, *r* = 0.99, *p* < 0.01 for 2014 and *r* = 0.98, *p* < 0.01 for 2015)^26,49–53^. For any given elevation, soil moisture is always higher in the Hii River, owing to the smaller particle size of the riparian soil.

Unlike soil moisture content, the sampled riparian soil TN and TP concentrations did not show a specific trend with respect to surface elevation (Arakawa River, *r* = 0.4, *p* = 0.12 for TN and *r* = 0.35, *p* = 0.18 for TP; Hii River, *r* = −0.16, *p* = 0.6, for TN and *r* = 0.33, *p* = 0.2 for TP). The average riparian soil TN concentration at the Arakawa River site was lower than that at the Hii River. At the Arakawa River site, soil TN values ranged between approximately 0.1% and 0.2%, regardless of elevation, whereas the values distributed widely between 0.1 and 0.4% at the Hii River site. These high values can be caused by the smaller particle size of the riparian soil at the Hii River. No significant difference was observed from the previous studies^17,26,49–53^.

Riparian soil TP was relatively similar in both rivers, ranging between 0.01 and 0.05%. The TN: TP ratio ranged from 1 to 4 at both river sites^54^.

### Species-specific distributional elevation and H_2_O_2_ concentration

**Figure 2:**
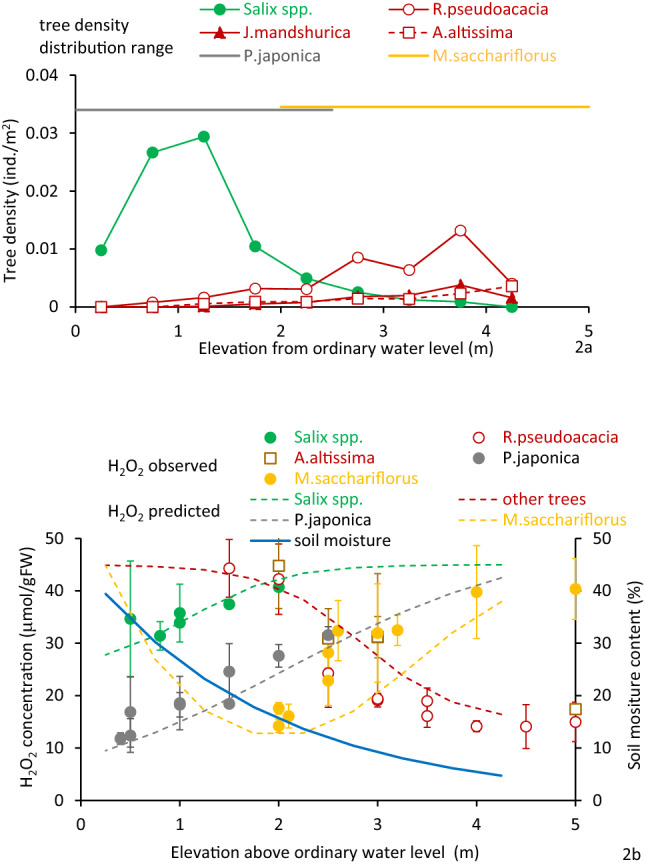
(**a**) Tree density and herb colonies at the Arakawa River site elevations of entire observed area. (**b**) Soil moisture and H_2_O_2_ concentration of samples and simulated results from Eqs. (3) to (6) distributions are also shown. Vertical bars in (**b**) are standard deviations.

**Figure 3:**
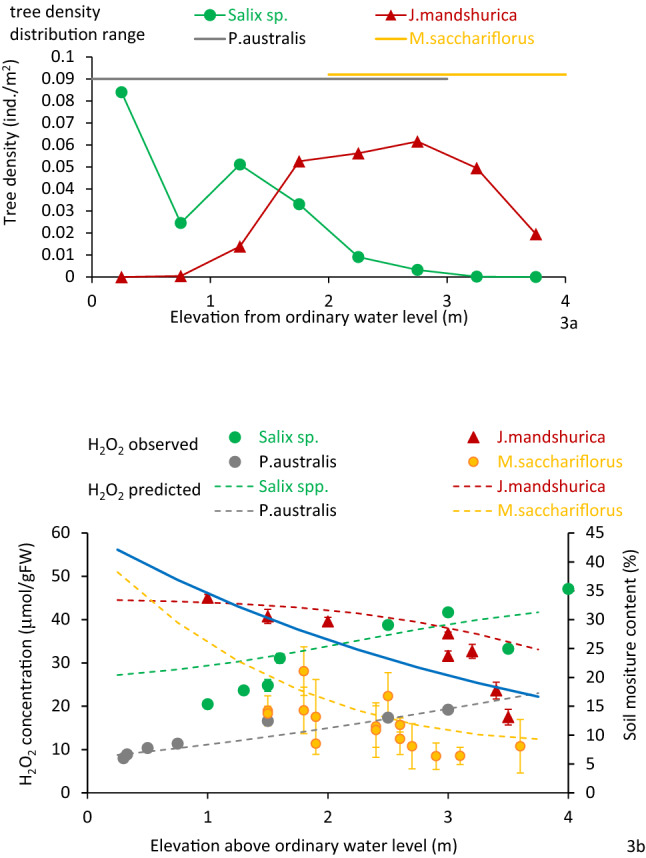
(**a**) Tree density and herb colonies at different elevations at the Hii River site of entire observe area. (**b**) Soil moisture and H_2_O_2_ concentration of samples and simulated results from Eq. (3) to (6) distribution are also shown. Vertical bars in (**b**) are standard deviations.

The elevation ranges, leaf H_2_O_2_ concentration of target species, and leaf H_2_O_2_ concentration compared with the soil moisture distribution are shown in Figs. 2 and 3. *Salix* spp. were distributed at elevations lower than 2.5 m from the ordinary water level at both river sites, while other tree species were distributed from 2.0 m upwards. For herbs, *Phragmite*s spp. were primarily located at elevations lower than 2.5 m from the ordinary water level, while *M. sacchariflorus* thrived from 1.5 m upwards.

### Foliar H_2_O_2_ concentration with respect to soil moisture content

**Figure 4:**
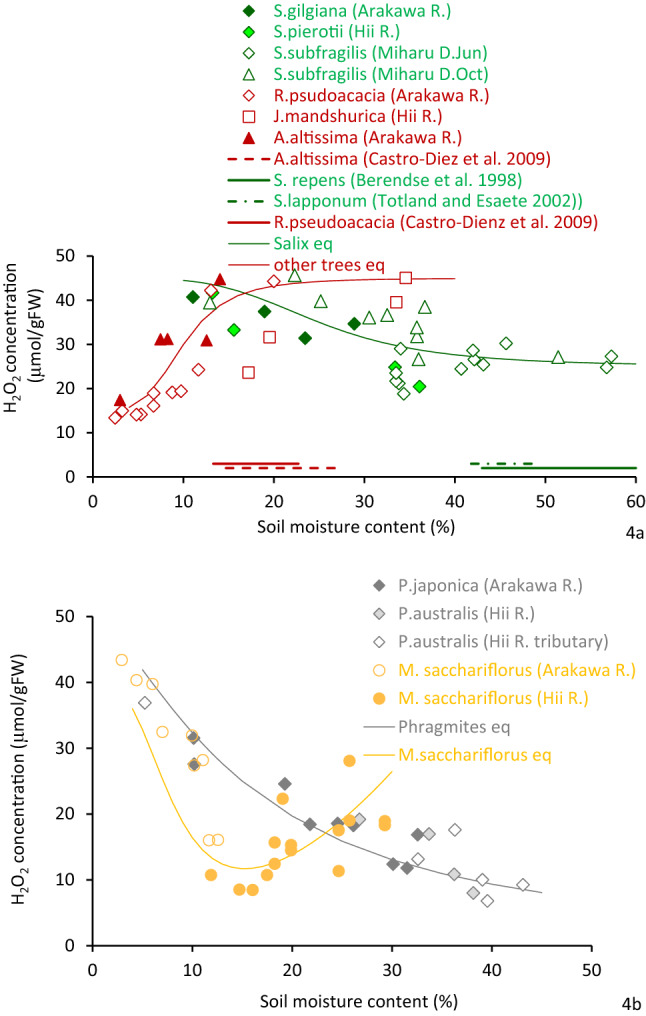
Relationship between soil moisture and leaf H_2_O_2_ for (**a**) different tree species and (**b**) herbaceous species. Reported soil moisture contents are added (Castro-Diez et al.^55^; Berendse et al.^56^; Totland and Esaete^57^).

Figure 4a displays the relation between soil moisture content and foliar H_2_O_2_ concentration of trees. The figure includes the foliar H_2_O_2_ values of *S. subfragilis* at the Miharu Reservoir^44^ and the soil moisture range of other reports^55,56^ for comparison. The *Salix* species localized generally at higher soil moisture sites than other species^57–61^. Regardless of rivers and the reservoir site, the foliar H_2_O_2_ concentration of all *Salix* species had a significant decreasing trend with increasing soil moisture content (*r* = −0.89, *p* < 0.01 for *S. pierotii*; *r* = −0.92, *p* < 0.1 for *S. gilgiana*; *r* = −0.5, *p* < 0.1 for *S. subfragilis*). This continued until the soil moisture content reached 35%, keeping the nearly constant value with higher soil moisture content. Thus, the lowest leaf H_2_O_2_ concentration (20 µmol/gFW) was recorded at higher than 35% soil moisture content.

Other tree species exhibited positive correlations between soil moisture content and H_2_O_2_ concentration (*r* = 0.66, *p* < 0.01). The value of H_2_O_2_ concentration (approximately 15 µmol/gFW) was lowest at around 5% soil moisture content (the driest condition), then rose with increasing soil moisture until approximately 20–30%, where it attained about 40 µmol/gFW.

Foliar H_2_O_2_ concentration of herbaceous species concerning soil moisture content is presented in Fig. 4b. Regardless of sites, similar species-specific trends were observed. *M. sacchariflorus* had a higher H_2_O_2_ concentration at low soil moisture conditions, which decreased with increasing soil moisture until 15% (*r* = −0.94, *p* < 0.001). It slightly increased again with higher moisture content. *Phragmites* spp. showed a uniquely decreasing trend with soil moisture content (*r* = −0.84, *p* < 0.001). Overall, there was a negative correlation between herb species and soil moisture content (*r* = −0.80, *p* < 10^−5^).

### Foliar H_2_O_2_ concentration with respect to other factors

TN and TP contents of plant biomass have organ-specific differences. Plant’s N: P ratios < 10 and > 20 often (not always) correspond to N- and P-limited biomass production in short term periods respectively, although it can vary in the long term^62,63^. The TN contents of plant biomass were approximately 0.9%, 0.4%, and 2.1% of roots, stems, and leaves of *Salix* spp., respectively, and 1.3%, 0.6%, 3.0% for *R. pseudoacacia*. TP contents were 0.1%, 0.01%, 0.12% for *Salix* spp. and 0.05%, 0.12%, and 0.07% for *R. pseudoacacia*. For herbs, whole-plant values were 1.9 ± 0.25% for TN and 0.19 ± 0.03% for TP. The nitrogen and phosphorus ratio of plant biomass’s range was 10 to 30. This was much larger than the nitrogen and phosphorus ratio of soils, which was 1 to 4. Therefore, compared with phosphorus, nitrogen seems to be more restrictive for plant growth. However, given the correlation between biomass (of *P. australis* or *M. sacchariflorus*) and riparian soil, the TN and TP contents were 0.24 ± 0.05 (*p* = 0.37) for TN and 0.1 ± 0.37 (*p* = 0.71) for TP (data not shown). There was no correlation between biomass and TN or TP contents in the riparian soil.

**Figure 5:**
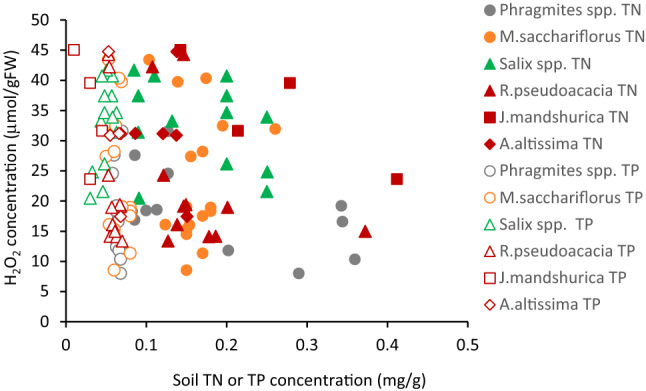
Relationship between leaf H_2_O_2_ concentration and soil TN or TP contents.

The H_2_O_2_ concentration with respect to riparian soil TN or TP contents is shown in Fig. 5. *J. mandshurica* were distributed at a wide range of TN concentrations, compared with other species, while *Salix* species’ habitat had relatively low riparian soil TN concentration. However, the H_2_O_2_ concentration of all species scattered largely, and there was no significant correlation with riparian soil TN or TP concentrations, and TN: TP (*S. gilgiana*: *r* = −0.16, *p* = 0.73 for TN and H_2_O_2_, *r* = −0.43, *p* = 0.34 for TP and H_2_O_2_, *r* = −0.06, *p* = 0.90 for TN: TP and H_2_O_2_; *S. pierotii*: *r* = −0.31, *p* = 0.55 for TN and H_2_O_2_, *r* = 0.66, *p* = 0.15 for TP and H_2_O_2_, *r* = −0.46, *p* = 0.36 for TN: TP and H_2_O_2_; *J. mandshurica*: *r* = −0.80, *p* = 0.1 for TN and H_2_O_2_, *r* = −0.78, *p* > 0.1 for TP and H_2_O_2_, and *r* = −0.13, *p* = 0.76 for TN: TP and H_2_O_2_; *R. pseudoacacia*: *r* = 0.35, *p* = 0.25 for TN and H_2_O_2_, *r* = −0.56, *p* = 0.08 for TP and H_2_O_2_, *r* = −0.256, *p* = 0.45 for TN: TP and H_2_O_2_; *A. altissima*: *r* = −0.187, *p* = 0.67 for TN and H_2_O_2_, *r* = −0.71, *p* = 0.178 for TP and H_2_O_2_, *r* = 0.25, *p* = 0.690, for TN: TP and H_2_O_2_).

For herb species, *M. sacchariflorus* were distributed over a relatively wide range of riparian soil TN concentrations (0.15–0.26%), and its foliar H_2_O_2_ concentration varied between 16 and 50 µmol/gFW. *P. australis* exhibited 12 to 32 µmol/gFW of H_2_O_2_ concentrations in 0.10–0.35% of riparian soil TN concentration. *P. japonica* were distributed near the shoreline of the channel, where TN concentration was relatively low. There was no significant correlation between H_2_O_2_ and TN or TP concentrations and TN: TP in riparian soil (*P. japonica*: *r* = −0.063, *p* = 0.871 for TN and H_2_O_2_, *r* = −0.497, *p* = 0.174 for TP and H_2_O_2_, *r* = 0.041, *p* = 0.917 for TN: TP and H_2_O_2_; *P. australis*: *r* = 0.454, *p* = 0.546 for TN and H_2_O_2_, *r* = −0.754, *p* = 0.246 for TP and H_2_O_2_, *r* = 0.748, *p* = 0.246 for TN: TP and H_2_O_2_; *M. sacchariflorus*: *r* = 0.044,* p* = 0.866 for TN and H_2_O_2_, *r* = −0.241, *p* = 0.352 for TP and H_2_O_2_, *r* = 0.181, *p* = 0.486 for TN: TP and H_2_O_2_).

The non-linear regression analysis was performed for each species to find out effect of different parameters (soil moisture, H_2_O_2_, TN, TP, TN: TP). *Salix* spp., *R*. *pseudoacacia*, *A*. *altissima*, and *J.mandshurica* individually show significant correlation between soil moisture content and H_2_O_2_ (*p* < 0.001, *p* < 0.001, *p* = 0.002, and p < 0.05 respectively), whereas no significant results were observed (*p* = 0.459 for *Salix* species, *p* = 0.884 for *R*. *pseudoacacia*, *p* = 0.186 for *A.altissima*, and *p* = 0.652 for *J.mandshurica*) among parameters (soil moisture, TN, TP, and TN:TP). Herb species also exhibit a similar type of significant trend in the nonlinear regression analysis. *Phragmites* spp. and *M. sacchariflorus* indicate significant correlation between soil moisture content and H_2_O_2_ (*p* < 0.001 for both species) On the contrary, no significant results were noticed (*p* = 0.076 for *Phragmites* spp., and *p* = 0.138 for *M. sacchariflorus*) among parameters (soil moisture, TN, TP, and TN:TP).

We cannot find significant trends between H_2_O_2_ concentration and riparian soil TN or TP. One of the reasons is because the variation range of TN and TP was too small to affect H_2_O_2_ concentration in the observed sites^64^, indicating the insignificant effect of nutrients on H_2_O_2_. However, widely various TN or TP variations may have a possibility to affect H_2_O_2_ concentration.

### Modeling foliar H_2_O_2_ concentration

From the above discussion we can conclude that due to the insignificant impact of nutrients especially TN or TP concentration, the H_2_O_2_ concentration in plants is solely related to soil moisture content. The simple empirical equations can be formulated based on the Monod equations. The equations show the fundamental structure as easily explicit and broaden the underlying mechanism of the species.

For tree species:

*Salix* spp.

3 $${\text{H}}_{{2}} {\text{O}}_{{2}} \left( {\upmu {\text{mol/gFW}}} \right) = 20.0\frac{{25^{4} }}{{25^{4} + {\text{Soil}}\,{\text{Moisture}}^{4} }} + 25.0$$

(for the range of soil moisture < 60%, *r* = −0.60, *p* < 10^−4^).

Other tree spp. (*Juglans mandshurica*, *Robinia pseudoacacia*, *Ailanthus altissima*):

4 $${\text{H}}_{{2}} {\text{O}}_{{2}} \left( {\upmu {\text{mol/gFW}}} \right) = 30.0\frac{{{\text{Soil}}\,{\text{Moisture}}^{4} }}{{25^{4} + {\text{Soil}}\,{\text{Moisture}}^{4} }} + 15.0$$

(for the range of soil moisture < 40%, *r* = 0.76, *p* < 10^−4^).

For herbaceous species:

*Phragmites* spp.

5 $${\text{H}}_{{2}} {\text{O}}_{{2}} \left( {\upmu {\text{mol/gFW}}} \right) = 50.0\frac{{15^{1.5} }}{{15^{1.5} + {\text{Soil}}\,{\text{Moisture}}^{1.5} }}$$

(for the range of soil moisture < 45%, *r* = 0.92, *p* < 10^−9^).

Miscanthus sacchariflorus:

6 $${\text{H}}_{{2}} {\text{O}}_{{2}} \left( {\upmu {\text{mol/gFW}}} \right) = 45.0\frac{{8^{3} }}{{8^{3} + {\text{Soil}}\,{\text{Moisture}}^{3} }} + \frac{{{\text{Soil}}\,{\text{Moisture}}^{2} }}{35.0}$$

(for the range of soil moisture < 30%, *r* = 0.89, *p* < 10^−3^);

 where soil moisture is given by %.

**Figure 6:**
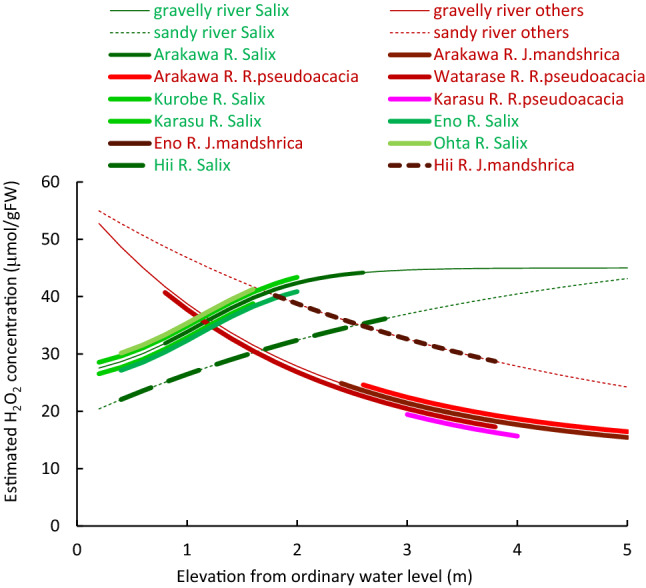
Estimated H_2_O_2_ concentration of tree species as a function of elevation from ordinary water level, compared with observed distribution (solid lines: gravelly reaches; dashed lines: sandy reaches; thick lines: observed distribution; green lines: *Salix* spp.; red lines: other species; Arakawa R., Hii R., Watarase R., Eno R., Ohta R.: this study; Karas R.^17^; Kurobe^27,66^).

**Figure 7:**
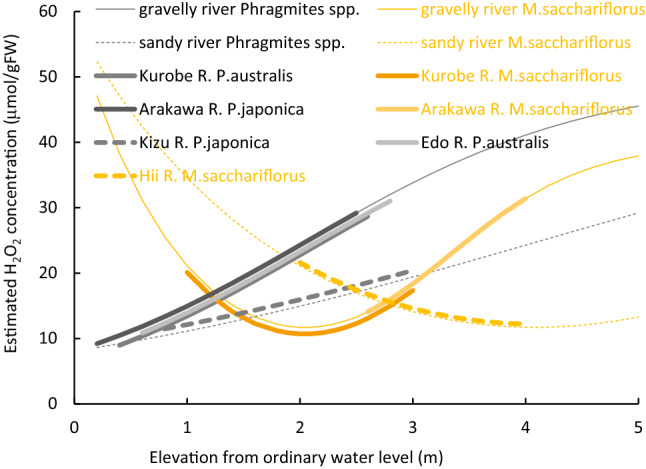
Estimated H_2_O_2_ concentration of herb species as a function of elevation from ordinary water level, compared with observed distribution (solid lines: gravelly reaches; dashed lines: sandy reaches; thick lines: observed distribution; gray lines: *Phragmites* spp.; orange lines: *M.sacchariflorus*; Arakawa R., Hii R., Watarase R., Edo R.: this study; Karas R.^51^; Kurobe R.^27,67^).

Soil moisture distribution depends on the sediment particle size composition. This relationship was similar for either gravelly or sandy rivers (Fig. 1). For simplicity, soil moisture at the ground surface at the Arakawa River site, Eq. (1), and the Hii River site, Eq. (2), are used as representative of those gravelly rivers and sandy rivers in general. Substituting Eqs. (1) and (2) into (3) to (6), H_2_O_2_ concentration for each type of plant is given as a function of the elevation. The estimated H_2_O_2_ concentration is presented in Figs. 6 and 7. The locations of observed plants in the present study and other reports are displayed by thick lines in the figures.

Both *Salix* spp. and other tree species are restricted to areas with stress levels less than 40 to 45 μmol/gFW. Therefore, *Salix* spp. can only distribute starting from the ordinary water level up to 4 m in gravelly rivers and 6 m in sandy rivers. In contrast, other tree species can distribute from 1 m upwards in gravelly rivers and 3 m upwards in sandy rivers. For herbaceous species, all species areas appear where soil moisture results in an H_2_O_2_ concentration below 40 μmol/gFW. *Phragmites* spp. can, therefore, distribute at elevations up to 3 m in gravelly rivers and more than 5 m in sandy rivers. *M. saccharifloru*s grows at higher elevations and cannot grow below1.5 m above the ordinary water level.

**Figure 8:**
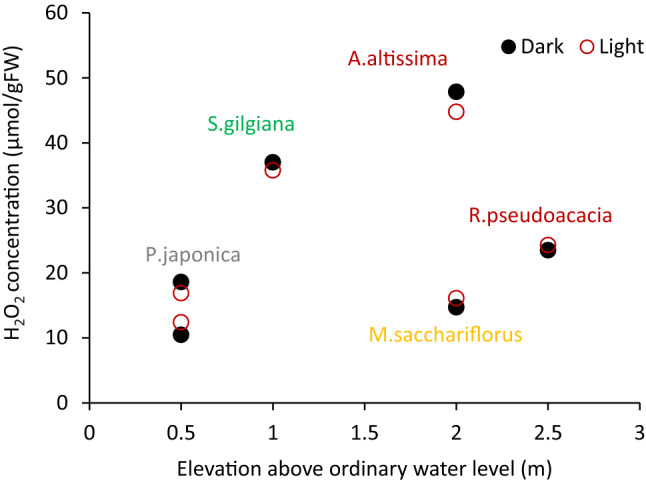
H_2_O_2_ concentration differences of light-exposed and dark-adapted samples.

H_2_O_2_ is generated under stressed conditions. Thus, the intensity of stresses is correlated to the H_2_O_2_ concentration. H_2_O_2_ concentrations of light-exposed and dark-adapted samples are shown in Fig. 8. There was no difference in H_2_O_2_ concentration between the two treated leaves (*p* = 0.96) for all tested species, indicating that dark conditions do not cause significant stress.

To ensure the efficacy of our derived equation, we try to analyze their suitability with the general additive model (GAM). We found that GAM supports our derived equations appropriately among different parameters (elevation, soil moisture content, H_2_O_2_, TN, TP, and TN: TP). The H_2_O_2_ concentration of tree species increased with higher soil moisture content, yet in terms of *Salix* spp., the relation is vise-versa. In the case of herb species, *Phragmites* spp. shows a significant negative correlation with soil moisture content and H_2_O_2_, whereas *M. sacchariflorus* shows a “U” shaped correlation with soil moisture content and H_2_O_2_. Considering these situations and derived equations, tree and herb species were divided into four groups in GAM analysis. Twso groups from tree species (*Salix* spp. and other tree species), and another two groups from herb species (*Phragmites* spp. and *M. sacchariflorus*). *Salix* spp., *Phragmites* spp., *M. sacchariflorus*, exhibit significant correlation with soil moisture content and H_2_O_2_ among parameters (elevation, soil moisture content, H_2_O_2_, TN, TP, and TN: TP) (Tables 1, 2 and 3). In the case of other tree species, significant correlation occurs with H_2_O_2_ and soil moisture content shown in Table 4, and elevation shows a strong correlation with H_2_O_2_ (Table 4). Supplementary Fig. 1 and Supplementary Fig. 2 represent the observed correlation with confidence intervals.

**Table 1:** General additive model for *Salix* spp. to find out correlation among parameters.

Parameters	edf	Ref.df	F	p value
**Approximate significance of smooth terms**				
s(Elevation)	1	1	0.41	0.53735
s(Soil Moisture)	1	1	14.230	< 0.05
s(Soil TN)	1	1	0.001	0.97290
s(Soil TP)	1	1	0.359	0.56564
s(Soil TN: TP)	1	1	0.005	0.94761

R-sq.(adj) = 0.766 Deviance explained = 85.6%.

GCV = 20.288 Scale est. = 11.593.

**Table 2:** General additive model for *Phragmites* spp*.* to observe correlation among parameters.

Parameters	edf	Ref.df	F	p value
**Approximate significance of smooth terms**				
s(Elevation)	1	1	0.422	0.5368
s(Soil Moisture)	1	1	11.343	< 0.05
s(Soil TN)	1	1	0.054	0.8232
s(Soil TP)	1	1	0.309	0.5958
s(Soil TN: TP)	1	1	0.063	0.8090

R-sq.(adj) = 0.897 Deviance explained = 94%.

GCV = 8.8054 Scale est. = 4.7414.

**Table 3:** General additive model for *Miscanthus sacchariflorus* to find out relationship among parameters.

Parameters	edf	Ref.df	F	p value
**Approximate significance of smooth terms**				
s(Elevation)	1	1	0.001	0.9762
s(Soil Moisture)	1.981	1.998	6.642	< 0.05
s(Soil TN)	1	1	1.216	0.2988
s(Soil TP)	1.574	1.812	1.077	0.4555
s(Soil TN: TP)	1.290	1.490	0.779	0.3683

R-sq.(adj) = 0.864 Deviance explained = 92.2%.

GCV = 29.554 Scale est. = 15.916.

**Table 4:** General additive model for other tree species to evaluate interaction among parameters.

Parameters	edf	Ref.df	F	p value
**Approximate significance of smooth terms**				
s(Elevation)	1.980	1.998	10.618	< 0.01
s(Soil Moisture)	1	1	7.231	< 0.05
s(Soil TN)	1	1	0.639	0.431164
s(Soil TP)	1.606	1.844	2.273	0.078004
s(Soil TN: TP)	1	1	0.415	0.525103

R-sq.(adj) = 0.728 Deviance explained = 78.2%.

GCV = 45.069 Scale est. = 35.012.

## Discussion

In Japanese rivers, the volume of sediments has substantially decreased over the last five decades because of the construction of dams upstream, gravel mining, and the reduction of sediment inflow due to the afforestation of the upstream mountainous areas. Therefore, the riparian morphology and sediment characteristics remain relatively stable, even though it is subjected to recurrent large flooding events^17,27,65,66^. Most of the sampling points were inundated only during a large flooding event, which occurs approximately once every 10 years, with the inundation lasting for a maximum of one week. Therefore, the measured soil moisture content is characteristic of the study site most of the time. The moisture content that the riparian zone reaches is highly affected by sediment particle composition; it is higher at sandy channels than at gravelly channels, as shown in the present study. There were good agreements between the results of previously observed cases (from Eqs. (1) and (2)) and the current study at both river sites.

Several other factors can affect riparian vegetation stress. In the riparian zone of the observed reach, generally nitrogen rather than phosphorus becomes critical for plant growth^17,67–69^, and it may thus influence the growth of vegetation via soil moisture. However, in the present study, there was no correlation between plant tissue H_2_O_2_ concentration and TN along with TP concentration in the riparian soil for the tree and herb species as shown in Fig. 6. Therefore, nitrogen and phosphorus were considered as neither major factors for limiting growth nor regulating species distribution in both rivers. Also, solar radiation did not affect the H_2_O_2_ concentration as expressed in Fig. 5. The last major flooding was more than two years ago, and no erosion or deposition trace was observed on the soil surface^26^. Thus, mechanical disturbances on the ground surface during floods likely did not affect the distribution of plants. Therefore, soil moisture seems to be the major component of stress for the plants in this case.

### Threshold H_2_O_2_ concentration 

When a plant is exposed to environmental stress, it generates H_2_O_2_ in different organelles. This occurs partly in the scavenging process of various reactive oxygen species and partly directly^37,38^. However, some H_2_O_2_ is scavenged by the antioxidant activities and finally converted to water and oxygen. Under high-stress conditions, plant tissues become damaged and die off due to the high concentration of H_2_O_2_. Therefore, there is likely a threshold concentration below which plants do not deteriorate or die^47,48^. In the present study, no cases with H_2_O_2_ concentrations higher than 40–50 µmol/gFW were observed for tree species. Concentrations above 40 to 50 µmol/gFW of H_2_O_2_ seem to be too high to maintain a healthy condition for a tree species. For example, *Salix subfragilis* in the Miharu Dam highly deteriorated at H_2_O_2_ concentration levels ~ 40 to 50 µmol/gFW, which was caused by at least 5 months of low soil moisture^44^. The low soil moisture condition seems to be a high stressor for *Salix* spp. At the same time, each species distributed only the elevation where the H_2_O_2_ concentration becomes lower than these values.

In the present study, *R. pseudoacacia* and *A. altissima* growing on low elevation sites along the Arakawa River had a high H_2_O_2_ concentration of 40 to 50 µmol/gFW. These plants may experience greater stress with high soil moisture. All *R. pseudoacacia* and *A. altissima* plants intrude and grow at the lower elevation sites (below 1 m, 30% moisture; Fig. 1), had died off over the course of several years^26,52,54^ without being subjected to flood disturbance. This situation indicates that more than 40–50 μmol/gFW of H_2_O_2_ concentration reflects harsh conditions for *R. pseudoacacia* and *A. altissima* (Fig. 2a). In contrast, all *S. gilgiana* growing at elevations above 3 m had disappeared after several years^26,52,54^. *S. gilgiana* cannot spread at this elevation, probably because of very low soil moisture content. For herbaceous species, no *Phragmites* spp., were found at elevations with less than 10% soil moisture in the gravelly channel and 20% in the sandy channel. This corresponds to an H_2_O_2_ concentration of 35 μmol/gFW and above. *M. sacchariflorus* distributed at relatively low soil moisture, with the lowest being about 5%, corresponding to higher than 40 μmol/gFW of H_2_O_2_ concentration. Although the water potential was not measured in this study, the sediment’s logarithmic scale of suction (pF) was approximately 4 at 10% moisture content^69,70^. The pF ~ 3 to 4 corresponds to the wilting point and is nearly the critical condition for the continued growth of many herbaceous plants^71,72^. The 40–50 μmol/gFW of H_2_O_2_ concentration obtained for the existing plants seems to be the critical value for survival. The lowest elevation where *M. sacchariflorus* was found was approximately 1.5 m above the ordinary water level, where the plant had about 30 μmol/gFW of H_2_O_2_ concentration. The location seemed close to the lowest elevation for the distribution, although this was not absolutely critical. Thus, the H_2_O_2_ concentration of *M. sacchariflorus* is U-shaped with respect to soil moisture. For other species, *Salix* spp. and *Phragmites* spp. were not found in water, and the H_2_O_2_ concentration is unknown, and other tree species growing at very low soil moisture conditions were not obtained. However, there is a possibility the H_2_O_2_ concentration also has a U-shaped trend.

The threshold condition of H_2_O_2_ concentration spans a relatively wide range, from 35 to 50 μmol/gFW species specifically. However, plants begin to die off with H_2_O_2_ concentrations higher than the threshold value as was observed in this study. Thus, the threshold condition delineates the habitat conditions where plants can form.

### Characteristics of tree species

*Salix* species usually are dominant at low elevation zones^57–61^. In the present study, *Salix* species always developed above the ordinary water level, and no *Salix* was found in the constantly inundated zone. *Salix* spp. make use of hydrochory: dispersal of seeds through water, which encroaches onto the shoreline in spring^44,58^. Therefore, the distribution of *Salix* spp. seems to reflect the physiological survival of occupied seeds.

For *Salix* spp., H_2_O_2_ concentration decreases accordance with soil moisture content till 40%. This situation indicates low stress in high soil moisture content. Then, it slightly increased with more than 40% soil moisture. The preferable soil moisture of these *Salix* species appears to be around 40%, which was near saturation. Several experiments indicated that even steady inundation does not lead to high stress as long as oxygen concentration is sufficiently present in the substrate^61^. Other tree species in the present study provided a relatively unique increasing trend of H_2_O_2_ concentration from 5% of soil moisture to higher levels. The soil moisture thus is the major component determining the potential locations for spatial distribution in the riparian zone.

For *R. pseudoacacia*, the highest photosynthesis rate was reported at 17% soil moisture. It was substantially lower at 8% and 24%^73^, and the highest reported leaf water potential was at 13% soil moisture, compared to lower soil moisture contents^74^. These results suggest the species’ preferred specific soil moisture conditions, which is confirmed in the present results (Figs. 2a and 3a). The soil moisture content at the root zone of trees was not measured in the present study. However, it can be derived from the soil moisture distribution in Fig. 1. The soil moisture at the deepest root zone (i.e., 4 m above the ordinary water surface and 2.5 m below the ground surface) was 16%, although the surface soil moisture content was ~ 5%^52^. This species does not seem to prefer a high moisture level, although it is typically found in riparian zones. Specifically, this species can thrive at higher elevations in the riparian zone^54,75^. Since this species uses hydrochory like the *Salix* spp., these results also imply that seeds can reach the preferred elevations only during very high water levels. *A. altissima* showed a similar trend of H_2_O_2_ concentration in response to soil moisture content as found in *R. pseudoacacia*. The H_2_O_2_ concentration level of *J. mandshurica* was slightly lower than *R. pseudoacacia* and *A. altissima*, indicating that this species prefers higher moisture content. Its high buoyancy can be more easily dispersed downstream and captured ashore at the riverside, where the soil moisture content is more congenial to its growth.

### Characteristics of herbaceous species

H_2_O_2_ concentration in herbaceous species decreased with increasing soil moisture, and this relationship was consistent regardless of species. *M. sacchariflorus* is one of the most common species of the riparian zone and is distributed at higher elevations than *Phragmites* spp. However, the H_2_O_2_ concentration of *M. sacchariflorus* was as high as 40 μmol/gFW when it grew on soil with approximately 5% moisture content. No distribution was found at higher elevations where the riparian soil has less than the soil moisture content. *Phragmites* spp., on the other hand, is typically distributed in the lower elevation zone where the soil moisture content is > 10%^16^. The tissue H_2_O_2_ concentration of *Phragmites* spp. in response to soil moisture content also indicates its preference for higher moisture conditions.

### H_2_O_2_ concentration as the monitoring system

Riparian plants are subject to various stress factors, and they establish successfully only in areas where stress levels are sufficiently low. The riparian zone is often affected by flood disturbance, which changes the channel topography. However, although the reduction of sediment flux erodes the channel bed, it provides a stable habitat for riparian vegetation^65,66^. Groundwater level variation takes a longer period than surface water and does not change much during a short period. If the soil moisture distribution is properly maintained with an appropriate period of low flow, regardless of short flood occurrences, the elevations where these species distribute or disappear can be predicted. Measurement of H_2_O_2_ can be a method to evaluate the threshold values of certain determining abiotic variables beyond which plants cannot survive. Therefore, their chance of disappearance under these conditions can be forecasted. This method is also more precise than most previous methods based on plant traits and empirical monitoring of their spatial distribution only. It is difficult to understand their preferred habitats by the general condition of their spatial distribution, owing to various types of stresses combined with different intensities and frequencies, and exposure periods^10,13,15^. Also, it is highly dependent on confounding factors like timing and unusual events. The physiological condition of each species determines their unique distribution, excluding physical mortality.

## Conclusion

Riparian vegetation encounters different environmental stresses. The findings of this study demonstrate that the spatial distribution of different species in a riparian zone occurs in the context of elevation and soil moisture content. The nutrient level especially TN and TP cannot play significant role due to a small variation of TN and TP concentration. The H_2_O_2_ concentration and soil moisture content solely significant in the observed sight. The species-specific distribution zones can be explained by the H_2_O_2_ concentration in the plant, which could be a rapid, efficient, and reliable monitoring indicator for vegetation distribution. This study suggests that foliar H_2_O_2_ concentration represents the sum of all abiotic stress, and the plant population decreases or cannot survive when it is beyond a critical value.

## Methodology

### Ethical permission

This study has been approved by the Ministry of Land, Infrastructure, Transport and Tourism, Japan (KasenGijutsuKaihatuH22-2, KasenBikaChousaH18-130, and KasenKikin 271271003).

### Study site

**Figure 9:**
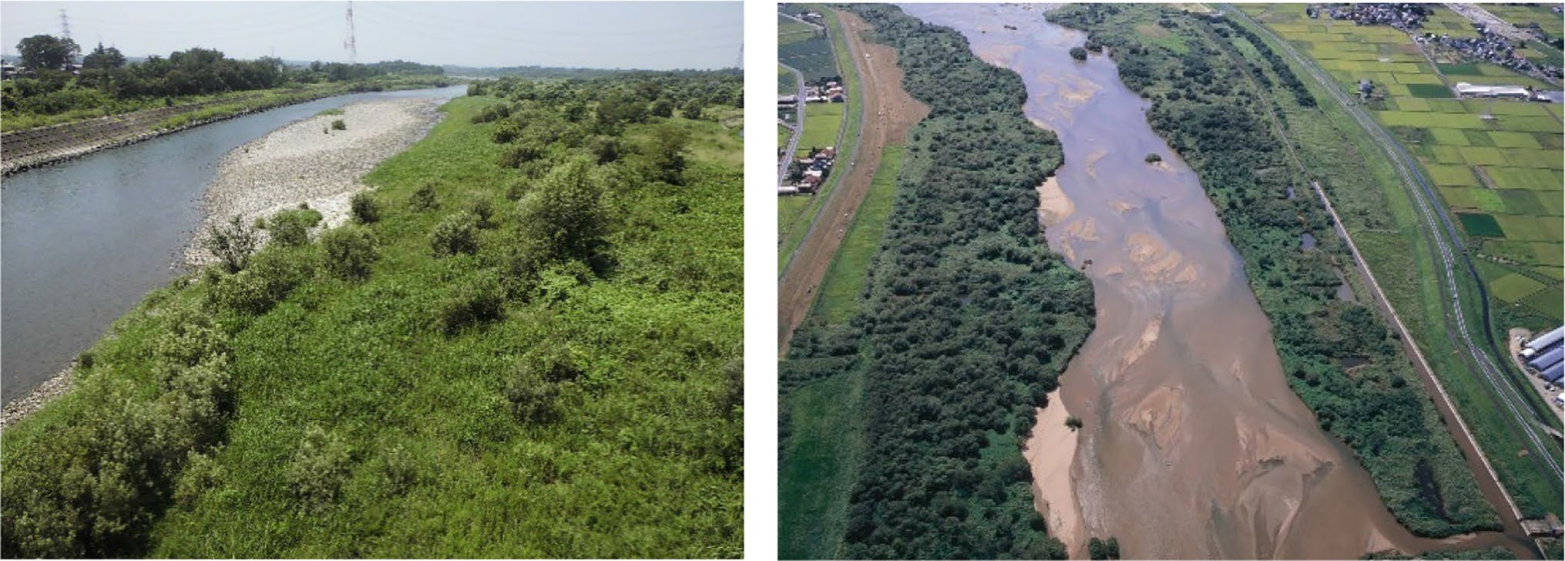
Studied sites at Arakawa River (left) and Hii River (right). Photo credits: Takashi Asaeda.

Field sampling was conducted in the gravelly sites of the Arakawa River and the sandy sites of the Hii River to compare differences depending on sediment particle size (Fig. 9). Both sites were in the anastomosing section. The Arakawa River is located at the center of Japan, originating from the Chichibu Mountains, flowing over 173 km before draining into Tokyo Bay. The middle section of the river consists of gravel channels typical of Japanese rivers.

Sampling was conducted at the Kumagaya Oaso gravel bar, 84 km upstream of the river mouth (36°8′20″ N, 139°20′35″ E). The channel slope was approximately 1/500, and the mean sediment particle size at the sampling site was approximately D_50_ = 50 mm (D_25_ = 10 mm, and D_75_ = 99 mm). The land use in the basin upstream of the sampling site was mostly agriculture and forests. Historically, the riparian zone was occupied by non-vegetated gravel areas until 50 years ago. However, vegetation coverage has gradually increased since then due to river regulation, and now half of the area is covered with woody or herbaceous plant species. More details of the Arakawa sampling site are given in other works of the authors^16,17,26,49,52^.

The Hii River is located in the Shimane prefecture (Western region of Japan). The river originates from the Chugoku Mountains and flows first into Shinji Lake, then into Naka Lake before draining into the Sea of Japan. It extends about 153 km and drains a basin of about 2017 km^2^. Eighty percent of the basin area is covered with forest, and the downstream 20% is covered chiefly with rice fields and residential areas. The sampling site was located 14 km upstream of Shinji Lake (35°21′57″ N, 132°47′20″ E), where the channel slope was approximately 1/600, and the bed was entirely composed of sand, D_50_ = 0.5 mm (D_25_ = 0.1 mm and D_75_ = 0.8 mm). This sand had been deposited in the past after iron mining activities upstream.

The riparian zones of both sampling sites are characterized by an upward elevation gradient across the river channel, reaching about 10 m in elevation above the water level in its ordinary flow condition. Groundwater levels have been relatively constant over the years and are tied to the ordinary river water level, at least at the locations where sampling was conducted (The Arakawa Upstream River Management Office, The Izumo River Management Office). Therefore, the riparian topsoil-to-groundwater distance increases as the distance from the river channel increases, while the overall soil moisture level decreases. The sediment’s rhizospheric zone was not saturated with water, and oxygen could penetrate sufficiently except in the zone close to channels^76,77^. All sampling points were taken on the riparian slopes where the ground surface was moderately covered with vegetation without being completely shaded. Both rivers and riparian zones are subject to intense flooding events. These floods are generally short and are caused by heavy rainfall. Once every 10 years, a major flood occurs. In all cases, however, the water level returns relatively quickly to the normal condition, that is, within less than a week. Except for these events, the water surface level is relatively stable. The soil moisture pattern in the riparian zone, which decreases with elevation from the ordinary water level, has not changed significantly for decades, as evapotranspiration rate balances with precipitation rate in most seasons. These conditions make the selected field sites excellent for studying the relationship between soil moisture and plant stress.

### Plant and soil sampling

Sampling was conducted between 10:00 and 14:00 on days with normal, fine weather conditions for the respective sites and more than a month after the most recent flood. In the Arakawa River, the sampling dates were May 5 and June 6, 2017, nearly 2 years after a large flood on September 9, 2015, which submerged sampling points. In the Hii River, sampling dates were October 11–13, 2016, more than 3 years after a large flood. The sampling dates were more than a year later than flooding in the respective rivers in all cases.

Dominant plant species of each site were sampled for this study. The major tree species at the Arakawa site were *S. gilgiana*, *S. subfragilis*, *R. pseudoacacia*, and *A. altissima;* the major herbaceous species were *P. australis*, *P. japonica*, and *M. sacchariflorus*. At the Hii River site, the major tree species were *S. pierotii* and *J. mandshurica*; the major herbaceous species were *P. australis* and *M. sacchariflorus*. After surveying 30 km of the riparian zone along the river reaches, three healthy mature individuals per tree species and five well-developed plants per herb species across the elevation gradient of the site were selected and marked.

Selected tree species were 3 to 5 m high, and herb species were about 1.5 to 2 m high. The average root depth of the sampled plants was approximately 2 ± 0.5 m for the tree species and 0.3 ± 0.1 m for the herb species (not sampled, data based on the works)^17,26,49,50,52^.

Well-grown leaves exposed to solar radiation were carefully sampled by hand. To measure the difference between the solar radiation exposed and dark-adapted samples, additional leaves of the tree species were shaded for 30 min by covering them with a well-ventilated paper bag before sampling. All sampled leaves were sealed in a plastic bag immediately after sampling and stocked in an icebox filled with dry ice (~ − 70 °C) to transport to the laboratory, where samples were frozen at − 80 °C until analysis. It took 8 h at most to transport to the laboratory. The half-life period of H_2_O_2_ is about 1.4 to 58 h in water^78,79^.

The low temperature in the stocked icebox may be a stress to changing H_2_O_2_ concentration. However, the temperature declined quickly to below − 10 °C, which is lower than the freezing point of H_2_O_2_ as the tissue thickness was less than 1 mm. There was no significant difference in the H_2_O_2_ tissue concentrations between the icebox-stocked and immediately analyzed samples (*p* = 0.76) and between icebox-stocked and non-stocked samples (*p* = 0.90).

The exact location of all sampled plants was recorded using GPS (Garmin eTrex). Absolute elevations of sampling points and the ordinary water level above the sea level (referenced to Tokyo peil (T.P.)) were obtained from the surveyed sectional map of the Upstream Arakawa River Management Office for the Arakawa river site and the River Management Offices of Izumo for the Hii River site. Soil samples were collected in triplicate from 20 cm below the ground surface from all plant sampling points. The riparian soil was stocked in tightly sealed plastic bags and brought to the laboratory for analysis.

### H_2_O_2_ assays of plant leaves

In the analytical process of H_2_O_2_ concentration, the fresh plant leaves were dry frozen with liquid nitrogen and ground (~ 500 mg) together with an ice-cold 50 mM phosphate buffer (pH 6.0). Polyvinylpyrrolidone (PVP) was added to this extraction to mask the effect of phenolic compounds in the plant materials. The extraction was centrifuged at 5000 rpm at 4 °C for 15 min, and the supernatant was separated. An aliquot of 750 μL extracted supernatant was mixed with 2.5 mL of 0.1% titanium sulfate in 20% (v/v) H_2_SO_4_^80^. The mixture was centrifuged at 5000 × *g* at 20 °C for 15 min. The intensity of the yellow color developed through the reaction was measured spectrophotometrically (UV mini 1210, Shimadzu, Japan) at a wavelength of 410 nm. The absorption at 410 nm includes the effect of other soluble compounds^81^. Thus, the H_2_O_2_ concentration was calculated from the slope of the standard curve obtained from the known H_2_O_2_ concentration, which was offset, derived by the intercept absorption rate with zero H_2_O_2_ concentration samples^82^. The results were compared with those of the e-FOX method^68^, and a suitable correlation (*r* = 0.971) was obtained. The results were presented as μmol/gFW (Fresh weight).

### Soil analyses

The moisture content of the soil samples was determined using the weight loss method. The soil sample was weighed initially and subjected to drying at 105 ºC in an electric oven until a constant weight over time was recorded. Soil moisture content was estimated by the difference between the initial and final weights of the sample. Soil nutrient analyses were conducted only for the fine sediment component (< 128 μm). TN and TP were analyzed separately from the soil in different method. TN in the sediment (oven-dried) was analyzed by a CHN elemental analyzer (Yanako CHN coder MT-5 and Autosampler MTA-3, manufactured by Yanako CO., Ltd., Japan). TP was determined by the molybdenum blue colorimetric method^83^ after digestion with H_2_SO_4_–HClO_4_ (APHA, 1998)^84^.

### Distribution analysis of target species

In both river sites, all individual trees taller than 1 m were counted per species for the entire gravel bar for the Arakawa River and the area between the dikes of the Hii River along a 2.7 km long stretch^85^. Compared with the channel morphology obtained by the MLIT River Management Office of each river, the Arakawa Upstream River Management Office, and the Izumo River Management Office, tree densities were obtained for every 0.5 m elevation.

### Statistical analysis

Data analyses were carried out using R^86^ for the dependent variables. Raw data of all variables were checked for normal distribution with the one-sample Kolmogorov–Smirnov test and for homogeneity of the variances with Levene's test. When necessary, arcsine transformation was performed. Results are presented as mean ± SD (n = 3). Data were subjected to a one-way Analysis of Variance (ANOVA) followed by Dunkan’s multiple range test to evaluate the mean difference at a 0.05 significance level. The correlation coefficient (*r*) and the significance levels (*p*) were used to evaluate the strength and significance of the parameters estimated. Non-linear regression analysis was performed to view species to species effect among parameters (H_2_O_2_, soil moisture content, TN, TP, and TN: TP). A general additive model was performed to find out the efficacy of derived Monod equations among parameters (soil moisture, H_2_O_2_, TN, TP, TN: TP, and elevation).

## Supplementary Information

Supplementary Figures.

Supplementary Information 1.

## Data Availability

The authors highly appreciate and state that data is available for everyone in the supplementary file named Raw Data.
