# Supplementary material for: Measurement of foliar H2O2 concentration can be an indicator of riparian vegetation management
Source: Sci Rep. 2022 Aug 13;12:13803. doi: 10.1038/s41598-022-17658-2 (PMC9376084; doi:10.1038/s41598-022-17658-2)
Supplement: Supplementary file 1 — Supplementary Figures. [file 41598_2022_17658_MOESM1_ESM.docx]

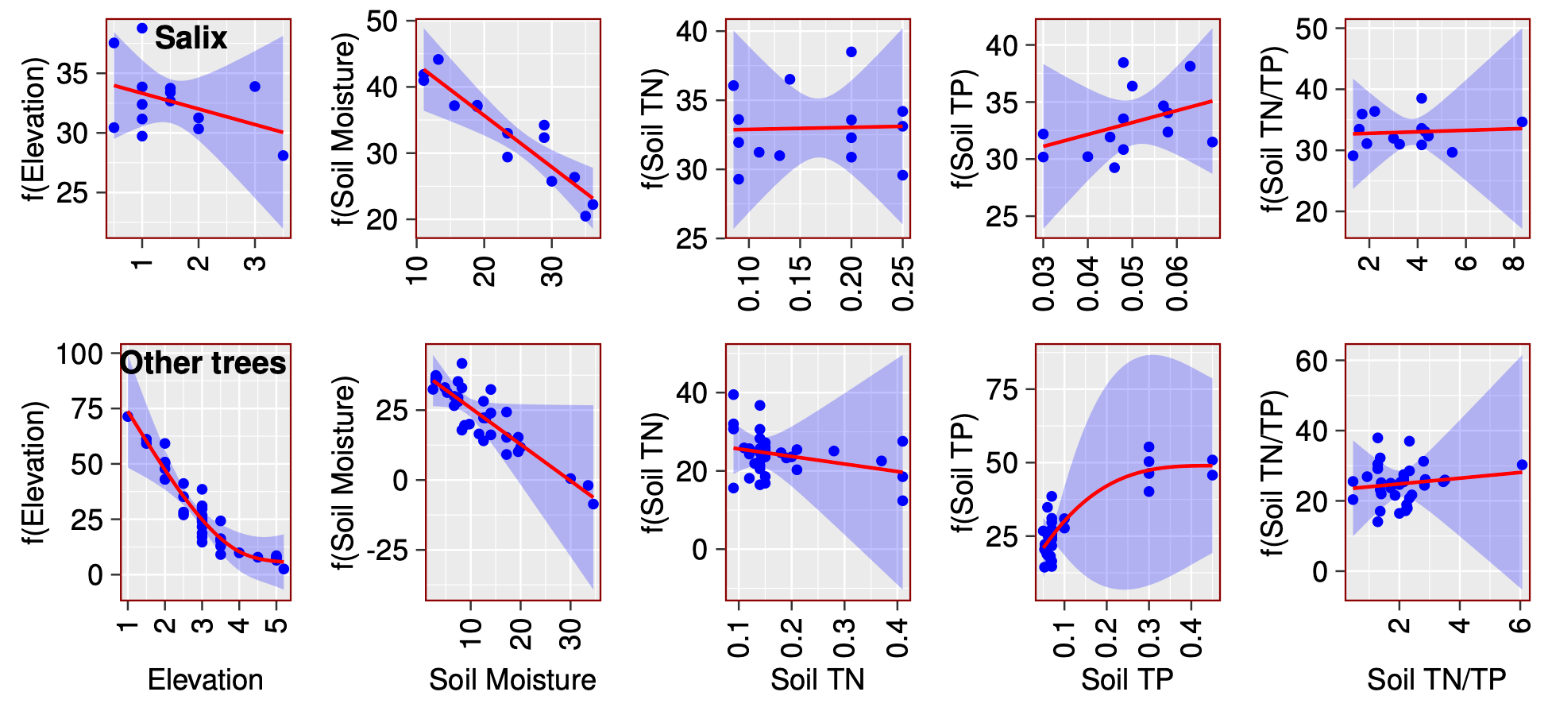


Supplementary Figure. 1: Observed correlation among parameters through GAM with tree species


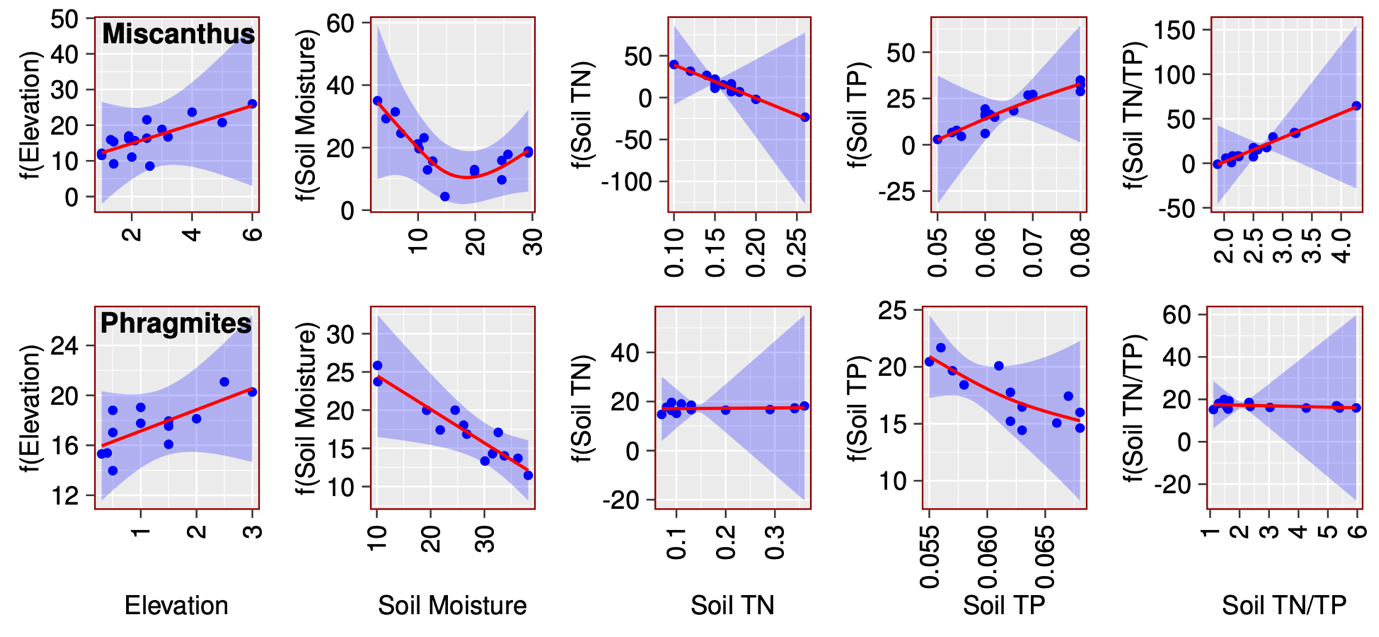


Supplementary Figure. 2: Observed correlation among parameters through GAM with herb species
